# Cancer

**Published:** 1884-12-20

**Authors:** 


### CANCER.

We feel it due to Dr. Lawson, the author of the case of Epithelioma in this number of the RECORD, to ask special attention to the article. In our April number he described a method of using arsenic, *which will cure cancer*, and reported a number of cases illustrative of his plan of cure. He now submits an additional case in verification of his discovery. The remedy seems heroic and hazardous ; but he assures us it is safe, as used by him. Its apparent danger should not debar its trial on a disease which, thus far, has defied all efforts for relief, and has become an *opprobium medocorun.*

A disease so horrible, and which has proven so invariably fatal, may well justify heroic, and even hazardous, measures, if thereby a cure be effected.

If the discovery made by Dr. Lawson had been announced by Koch, Pasteur, or other learned *savant* in some foreign land, it would probably have been eagerly seized upon by every journal in the country as an item of great interest and importance ; but coming, as it does, from a modest though worthy practitioner in the State of Georgia, it has failed, so far as we know, to attract the attention of any one, or to elicit a single comment by the medical press throughout the country.

### METRIC SYSTEM.

The following is the bill pending in Congress for the adoption of the Metric System, and will probably become a law :

A BILL to establish the metric system of weights and measures in the Departments of the Government :

Be it enacted by the Senate and House of Representatives of the United States of America, in Congress assembled, That, from and after the fourth day of March, anno Domini eighteen hundred and eighty nine, the metric system of weights and measures, as recognized and expressed in section thirty-five hundred and seventy of the Revised Statutes, shall be exclusively employed by the several Departments and branches of the Federal Government in the affairs of the United States : Provided, that in all other transactions than those in which the United States is a party it shall be lawful to employ the weights and measures now in use.

SEC. 2. That a knowledge of the said metric weights and measures shall be taught in all the schools and colleges now under the control of the Federal Government or hereafter aided by it, or such knowledge shall be required to admission to the said schools and colleges.

SEC. 3. That all laws inconsistent herewith are hereby repealed.

